# Community Health Workers as Influential Health System Actors and not "Just Another Pair Of Hands"

**DOI:** 10.34172/ijhpm.2020.58

**Published:** 2020-04-27

**Authors:** Sumit Kane, Anjali Radkar, Mukta Gadgil, Barbara McPake

**Affiliations:** ^1^Nossal Institute for Global Health, Melbourne School of Population and Global Health, The University of Melbourne, Parkville, VIC, Australia.; ^2^Gokhale Institute of Politics and Economics, Pune, Maharashtra, India.; ^3^State Health Systems Resource Centre, Pune, Maharashtra, India.

**Keywords:** Community Health Workers, Human Resources for Health, India, Performance, Low- and Middle-Income Countries

## Abstract

**Background:** Over the last 20 years, community health workers (CHWs) have become a mainstay of human resources for health in many low- and middle-income countries (LMICs). A large body of research chronicles CHWs’ experience of their work. In this study we focus on 2 narratives that stand out in the literature. The first is the idea that social, economic and health system contexts intersect to undermine CHWs’ experience of their work, and that a key factor underpinning this experience is that LMIC health systems tend to view CHWs as just an ‘extra pair of hands’ to be called upon to provide ‘technical fixes.’ In this study we show the dynamic and evolving nature of CHW programmes and CHW identities and the need, therefore, for new understandings.

**Methods:** A qualitative case study was carried out of the Indian CHW program (CHWs are called accredited social health activists: ASHAs). It aimed to answer the research question: How do ASHAs experience being CHWs, and what shapes their experience and performance? In depth interviews were conducted with 32 purposively selected ASHAs and key informants. Analysis was focused on interpreting and on developing analytical accounts of ASHAs’ experiences of being CHWs; it was iterative and occurred throughout the research. Interviews were transcribed verbatim and transcripts were analysed using a framework approach (with Nvivo 11).

**Results:** CHWs resent being treated as just another pair of hands at the beck and call of formal health workers. The experience of being a CHW is evolving, and many are accumulating substantial social capital over time – emerging as influential social actors in the communities they serve. CHWs are covertly and overtly acting to subvert the structural forces that undermine their performance and work experience.

**Conclusion:** CHWs have the potential to be influential actors in the communities they serve and in frontline health services. Health systems and health researchers need to be cognizant of and consciously engage with this emerging global social dynamic around CHWs. Such an approach can help guide the development of optimal strategies to support CHWs to fulfil their role in achieving health and social development goals.

## Background

In 1990 Gill Walt, in the title of her book on community health workers (CHWs), wondered whether CHWs had become “just another pair of hands.”^1^ While the last three decades have seen CHW programs being established and scaled up across the world,^2^ her question lingers in the minds of researchers and practitioners working in global health. The question influences the burgeoning body of research on the performance of CHWs^3-6^; which suggests that CHWs’ performance and lived experience is greatly influenced by the context in which they operate.^2,7,8^ Literature indicates that three broad features of context interact with each other and with the CHW program to shape CHW’s performance and experience.^5,9-17^ (A) The first contextual feature relates to the state of the health system – its accessibility, performance and responsiveness. (B) The second contextual feature relates to the characteristics of the community being served; societal characteristics like social and gender norms determine what is considered as constituting socially ‘important work,’ and thus determine the extent to which communities appreciate and value the work being done by CHWs. (C) The third contextual feature – the state of economic development in a society, both scaffolds and interacts with the first 2 contextual features, to shape access to resources, opportunities and status, and determines CHW’s performance and their overall experience of being a CHW.

In these comprehensive accounts of the many ways in which context shapes CHWs’ performance and lived experience, a key narrative, in many ways reminiscent of Walt’s title, stands out. For instance, Maes and Ippolytos^11^ in their analysis of CHW programs in Mozambique, Ethiopia and Pakistan, argue that the contextual influences are such that “acceptance and discrimination coexist.” They recognise a “sense of inequity” and argue that a major aspect of the problem relates to CHWs being treated “as resources to be better exploited through technical quick fixes.” A recent six-country comparative study also found that a wide range of contextual influences intersect such that being a CHW is often a net disempowering experience, particularly for women CHWs.^18^ Similarly, research from India^13,19,20^ has demonstrated how the contradictions and tensions in the social, economic, and health system contexts operate to undermine CHWs’ experience and performance. Recent research from other parts of the world^21-23^ also points to this narrative. For instance, Steege et al argue that because CHWs “sit at the bottom of the health system hierarchy”; they are at the receiving end of unequal and gendered structural power relations which define the health sector and their societies.^23^ Schneider,^24^ in her recent synthesis, similarly argues that it is “necessary to ensure that CHWs do not become lowly players at the bottom of a health worker hierarchy, drawn into health facilities as an ‘extra pair of hands.’” Schneider expresses concern that “CHWs risk being drawn into health facilities as menial workers, especially if they are answerable to facility-based providers.”^24^

In this article, using the Indian CHW program (Box 1 provides an overview of the program – by far the biggest such program in the world)^25^ as a case study, we provide a textured understanding of the experience of being a CHW. We show that far from being passive and at the receiving end of structural-contextual forces, increasingly, CHWs in India are actively negotiating, and covertly and overtly subverting the structural constraints they encounter, to fulfil their roles and also to serve their own advantage. We highlight and critically discuss the intentionality and deft exercise of agency by CHWs in navigating the challenging contextual environments within which they operate. We add to the knowledge base by demonstrating the dynamic and evolving nature of CHW programmes and the lived realities of being CHWs in low- and middle-income country contexts like that of India. We contend that as CHW programs in different parts of the world become mature and as CHWs establish themselves within communities, much can be gained from having a more nuanced understanding of CHWs’ evolving status and functioning within health systems and communities.

**Box 1. Community Health Workers in India**

With around a million CHWs, India’s ASHA program is the largest in the world – it reaches every village in the country. ASHAs are volunteers who receive a modest, activity linked, honorarium. ASHAs are all women; they are residents of the village they work in and are chosen and appointed by the village ‘Panchayat’ (the democratically elected village assembly). The ASHA program is the centrepiece of the National Rural Health Mission of India, the flagship public health reform initiative of the Government of India. The National Rural Health Mission, many of the national disease-specific programs, and other social welfare programs, increasingly call upon ASHAs to conduct and support their activities.

Abbreviations: CHWs, community health workers; ASHA, accredited social health activist.

## Methods

We draw upon data collected as part of a broader study which sought to answer the research question: How do accredited social health activists (ASHAs) experience being CHWs, and what set of factors shapes their experience and performance? To answer this research question, we conducted a qualitative inquiry with a two-pronged approach. Kane et al^6^ have postulated a set of 7 mechanisms which explain CHWs’ experience and performance; these postulates serve as the starting point of our inquiry. The extant literature on CHWs/ASHAs’ work and performance, our knowledge of the local social and health system context, our experience of working in the study area, and these postulates, informed the broader study. Further, to gain insight beyond what was articulated in the literature, an inductive approach was taken; the inquiry was informed by and continuously refined through a process of discussion and debate between the researchers, during data collection and the concomitant data analysis process. Supplementary file 1 presents the inquiry framework, and the interview topic guide.

 This article presents an interrogation of the data collected for the broader study with the aim of understanding ASHAs’ experience of being CHWs, and of revealing how ASHAs navigate the contextual and structural forces they are embedded in. Our intention is to spotlight ASHAs’ struggles, their agency, and to critically analyse their strategies to cope with, overcome and subvert the structural forces they encounter. Throughout we signpost implications for CHW programs, and for human resources for health policy and practice generally.

###  Study Context and Sites

We conducted the study in Pune district of Maharashtra State of India. Pune is a large district with 14 subdistricts and a population of 10 million. Approximately half of the population lives in the densely populated urban area, and the other in rural areas. Pune has a well-established, well-functioning public health service, with a good referral system which is well linked to high quality tertiary care facilities in urban Pune. We purposively selected 2 study sites in rural Pune; XX village in sub-district 1, and YY village in sub-district 2^[1]^. The 2 sites were similar in terms of health system characteristics and societal characteristics like language, culture, caste structure, social norms and gender norms. The 2 study sites were however different in terms of the state of economic development. XX village is a prosperous agriculturist community with superior quality lands. It is well-connected to the main road networks, and to Pune city; XX has good economic opportunities. On the other hand, YY village is a less prosperous community, is hilly, and has inferior quality lands. Transport infrastructure and connectivity with the main road network is good; economic opportunities are however limited.

###  Participants, Sampling and Recruitment 

We conducted interviews with purposively selected ASHAs and informants who could potentially shed light on the study subject from different perspectives. ASHAs were selected from among those who had their base at the primary health centre (PHC) at the 2 study sites – XX and YY village. We obtained the profile and contact details of ASHAs from the district coordinator of the ASHA program; the profiles contained information about age, marital status, education level, family, and number of years working as an ASHA. We (authors) met with ASHAs as a group at the 2 sites, interacted with them, and identified those who we judged would be able to provide us with rich insight. Appointments for interviews were made using mobile phones, and interviews were conducted at locations and at times that were convenient for the ASHA. Of the 75 ASHAs who were based at the 2 study sites, 17 were interviewed for the study; their profile is presented in Supplementary file 2.

We also interviewed the following key informants: ASHA supervisors (n = 4), nurse at the PHC (n = 2), multipurpose health worker (n = 2), doctor in charge of the PHC (n = 2), head of the village ‘panchayat’ (the village assembly) (n = 2), a family member of an ASHA (n = 2), and the district coordinator of the ASHA program (n = 1). Lines of inquiry included in the interview topic guide are presented in Supplementary file 1. Topic guides were developed in English and translated into Marathi, the local language. All interviews were conducted in Marathi and transcribed verbatim in Marathi. Informed written consent for participation was sought from and given by all study participants. Interviews were conducted until no new themes emerged – the point of saturation was identified during the debriefing and analysis sessions which we had at the end of each day of field work.

###  Analytical Approach

We used the framework approach^26^ to analyse our data. Analysis was done on an ongoing basis; each interview was analysed and discussed (at the end of each day) before proceeding further. This helped us to incorporate emerging themes and allowed the refinement and specification of existing themes in the topic guides and the coding frame. Ultimately a coding frame consisting of 22 codes was used. We coded the verbatim transcripts using NVivo 11 software; this was done in Marathi and the quotes used to illustrate the findings below, were translated at the time of inclusion in the article. Throughout this process we held regular discussions and often revisited the transcripts and/or the audio files. During these discussions we deliberated upon emerging themes and discussed potential theoretical frameworks which could help us refine the analysis. Our focus was on ‘interpreting’ and on developing analytical accounts; this exercise was thus iterative and occurred throughout the research: during the debriefings and discussions at the end of each interview, at the end of each day during the fieldwork, during our discussions on potential theoretical frameworks to help refine the analysis, during the process of coding of interview transcripts, and finally during the process of writing up emerging themes. While the interpretive element features throughout the findings and discussion sections below, our interpretive and analytical voice (as researchers) is occasionally made explicit.

## Results

In this section we present ASHAs’ experiences at work and in related social realms; we reveal the contextual and structural forces they are embedded within, and how they navigate these. Findings are presented in relation to 3 broad themes. In the first theme the focus is on ASHAs’ experiences as a result of their formal association with, and their ongoing regular interactions with the health system. In the second broad theme, ASHAs’ experiences as agents embedded within local social and gender relations are analysed. In the third theme we examine the findings to shed light on the tensions between the competing agency relationships ASHAs find themselves in the middle of. Throughout, we draw upon the 2 instances presented in Box 2 and Box 3, and on excerpts from interviews, to illustrate the findings.

**Box 2. Going the Extra Mile**

On being asked to share an example of a challenging situation she was faced with, an ASHA narrated the case of a pregnant woman who hailed from the poor, tribal ‘Katkari’ community, and lived in a remote hamlet in the hilly parts of YY village in sub-district 2. Because the pregnant woman was not feeling well, the ASHA urged her to go to the PHC and accompanied her there. At the PHC the woman was diagnosed as being severely anaemic and was advised to go to a higher-level facility. The ASHA could have simply asked the pregnant woman’s family to do this, but knowing the ‘Katkari’ community, it was obvious to her that if left to the woman’s family, no action would be taken, and the pregnant woman’s life would be in danger. The ASHA took the matter into her own hands and arranged for the pregnant woman to be taken to a tertiary hospital in Pune city. At the hospital, they were told that the patient required a blood transfusion urgently. At this point in the narration, the ASHA paused.

*“The interviewer probed and queried: So, what happened? The ASHA replied: They said she needs blood, and it costs Rupees 1200. Interviewer: So? ASHA: I promptly called the Medical Officer in Charge of the Sub-District … and told him that the patient is a ‘ Katkari ’ and cannot afford to pay … but that without the blood, her delivery won’t proceed. Sir (the Medical Officer in Charge of the Sub-District) urged me to pay for now, reassuring me that I would be reimbursed. Interviewer: He took the responsibility? ASHA: Yes, he did, and I paid from my own money. The doctors did a Caesarean Section and a baby boy was born. I went back to my village that evening but returned the next day. The doctors summoned me and said that someone needed to stay with the mother-child, to help with the food … to take care. I told them that she is a ‘ Katkari ’ and that it was unlikely that someone from her family would come. I called Sir again and got him to explain the situation to the hospital doctors. I also got the hospital to agree to give free food to the patient … for a week” [ASHA YY Village]*.

The ASHA went on to explain how she went further and organised the pregnant woman to receive the government financial incentives (approximately Rupees 1500) that she was entitled to by virtue of having delivered in an institutional setting.

Abbreviations: ASHA, accredited social health activist; PHC, primary health centre.

**Box 3. ASHAs as Skilled Social Actors**

In response to an open question about her experience of being an ASHA, one of the participants, in stating that her *“experience was very good,”* offered to share an instance of a recent case which she was involved in. She said *“this is a recent delivery, and they have really small babies at home … I will take you there (we visited the family).”* The ASHA explained that the woman started having labour pains in the seventh month of her pregnancy; the ASHA took her to the PHC and the doctors there advised the woman to go to a higher centre. The ASHA organised the public ‘108 Ambulance’ and accompanied the pregnant woman to the nearest tertiary referral hospital in the city (a private hospital).

There it transpired that it was twin pregnancy, the foetuses were underweight, the babies would be born premature, and would require special and expensive neonatal care. The costs of care in the private hospital would have been catastrophic (Rupees 500000) – the ASHA called the Sub-District Health Officer, and with the agreement of all, decided to move the woman to a public hospital. It was easier said than done – the ambulance was expensive, the patient had no money, and the private hospital would not offer the transport for free. Again, the ASHA called up an acquaintance in the Government’s Social Work Department and was able to arrange an ambulance for free; she accompanied the woman to a public hospital.

When the woman was taken to the delivery room it was found that she had her cervix stitched for cervical incompetence, to prevent miscarriage. The doctors at the public hospital could not remove the cervical stitches and referred her to the medical school for expert intervention. Again, the ASHA accompanied the woman to the medical school (name anonymised) and ultimately the twins were delivered.

But the struggle did not end there – the neonatal care unit was full, so the premature babies had to be referred out. The family could not afford it and without neonatal care unit care the babies would have died – once again the ASHA made calls to higher officers in the health services, social workers and doctors, pleading *“please … save our children … please do something,” *and somehow managed to continue care at Name Anonymised Hospital. The ASHA said, with a lot of pride in her voice that she *“spent 3 days away from home … running around … two days at Name Anonymised Hospital with the babies.”* She added with great satisfaction in her voice *“the woman’s family go around telling everyone what I did for them … they never tire of repeating how I saved their children’s lives. Their blessings will touch my children too … my family too … the satisfaction is immense” *[ASHA XX Village].

Abbreviations: ASHA, accredited social health activist; PHC, primary health centre.

###  Exercising Agency: Earning Credibility, Maintaining Legitimacy, Going Beyond the Call of Duty

Being linked to the local health services lent a sense of legitimacy and enhanced the feeling of credibility amongst the ASHAs. This overarching contextual influence had a wide ranging, positive influence on how ASHAs experienced being a CHW – in both professional and social realms. While all ASHAs emphatically reported that since becoming ASHAs, they had acquired greater respect from society, the instance in Box 2, and the excerpt below reveals the layered texture of the process of accrual of this credibility and legitimacy through ASHAs’ being consistently available, even in the middle of the night, being responsive, and going beyond the call of duty to help the villagers.

*“Interviewer: So, people regard ASHAs well? Respondent: People regard the ASHA highly. They see her as someone who works for the health of the villagers … see her as someone good. Interviewer: I see … OK. Respondent: Yes, they see her as someone who will rush to their assistance … say, if something happens to my pregnant daughter-in-law in the middle of the night. Such moments are critical … and in such situations (the ASHA will) mobilise an ambulance … even private vehicles.” *[ASHA XX Village].

 Recognition of the importance of being available to community members in times of need, was universally shared amongst the ASHAs. All ASHAs appreciated that being responsive was the only way to establish credibility and legitimacy in their community.

However, the association with the health system helped provide legitimacy primarily in the early stages of being an ASHA. For the more experienced ASHAs, the association seemed to matter less; over time, their legitimacy became a function of their credibility, which in turn was a function of how they performed and had performed. We noticed that ASHAs seemed to make a distinction between legitimacy and credibility. To them, acquisition of legitimacy was something that had occurred back in the past when they were initiated as ASHAs. The more established ASHAs we spoke to no longer seemed to need the association with the health system to legitimize their identities and their work. ASHAs across the 2 contexts had grown into and seamlessly assimilated into their roles; they seemed to effortlessly embody and confidently and effectively enact their roles, earning credibility in the process and cementing their status as legitimate social actors. In the excerpt below, a continuation of the conversation excerpted above, the health worker goes on to use the colloquial terms *‘ aapliye ’* and *‘ hakkachiye ’ *to refer to how the villagers have now come to view the ASHAs. The term *‘ aapliye ’* connotes someone who is seen as being close, and *‘ hakkachiye ’ *refers to someone who is seen as being so close that one can unhesitatingly call upon them at any hour, for any help – the connotation of credibility underpins both these terms. The ASHA highlights that people, including health workers, recognise that ASHAs have over time established strong local networks and accumulated relational capital, and that the situation now is that villagers turn to ASHAs when there is a health-related need, instead of turning to family and friends.

*“Interviewer: So, family members can’t, but ASHAs can? Respondent: (Yes, she knows) the network … that’s why. Interviewer: So, because she can do all this the villagers, the society … Respondent: because of all the work she does for health (of the villagers), people have trust in her. Interviewer: Ah … so how do the villagers view her? Respondent: Aapliye ho (One of their own) … Hakkachiye ” *[ASHA XX Village].

The instance in Box 2 is remarkable because the ASHA went out of her way for someone who hailed from the marginalized, tribal ‘Katkari’ community. The instance spotlights the ASHAs’ capacity to identify and articulate community needs in real time, and their potential to be active community advocates, including for those who are poor and marginalized.

 On the other hand, we observed that ASHAs across the 2 contexts, struggled, albeit quite successfully, to maintain their credibility. We found that whenever the health system was accessible, performed well, and was responsive (both to ASHAs’ expectations and to people’s expectations), it helped ASHAs maintain their credibility amongst community members. Across both the study sites, the health system sometimes fell short in terms of accessibility, performance, and responsiveness to ASHAs’ and people’s expectations – and it was during these times that ASHAs’ credibility amongst community members seemed to be most at risk. These shortfalls and failures annoyed and frustrated the ASHAs, because these failures were clearly beyond their influence. In both the study contexts, ASHAs devised sophisticated and nuanced strategies to uphold their credibility. For instance, wherever they sensed a failure developing, they worked extra hard to demonstrate to community members that it was happening despite them doing their best; sometimes this also entailed a bit of exaggeration and dramatization. For instance, we realised that when confronted with a failure of care provisions, ASHAs would occasionally resort to exaggerating the seriousness of a patient’s condition to the relatives; they would dramatize the complexity of the care needed and being provided. This seemed to serve at least 2 purposes – one, it served to catalyse the mobilisation and application of efforts and resources by the family, and second, the narrative served as a hedge or a risk management strategy in the not so unlikely event of the patient’s condition deteriorating.

ASHAs would do this with a lot of tact – always making sure that they were not seen as defending or justifying the system’s failures. At another level, as illustrated in Box 2 and Box 3, learning from these experiences, ASHAs devised strategies which involved mobilizing social and relational resources – from the local community and the health system, to overcome constraints and manage the failures. Instead of being cowed by the contextual forces (health system weaknesses in this instance), we found that ASHAs leveraged these failures to accrue social capital, and to maintain and enhance their credibility and their work. To us, the ASHAs seemed to be saying “As long as the system doesn’t mess up all the time or big time *…* we can handle it, and we can understand.” This pragmatism was deceptively simple; it reflected deft navigation of the many complex relationships that ASHAs had to manage across the community and the health system. It also showed that far from being the oppressed party, the ASHAs were skilfully and successfully tackling the adversities they encountered. The impression we came away with was that generally the ASHAs were able to overcome these adversities, including, as illustrated in Box 3 and discussed in the next section, through mobilising their social and professional networks.

 Nevertheless, and consistent with the excerpt below, all ASHAs acknowledged that the support they received in the early stages of their careers, from the health system and from community leaders, was critical in enabling them to gain confidence and in learning to navigate these complex relationships.

*“In the beginning I struggled. People would look at me and say … why is she doing this … how is it that she is telling us about health and giving us medicines? They would say … she isn’t a doctor … what does she understand? It was then that our village headman and the elders strongly stood by me. They told the villagers that I am an ASHA, and that I have the capacity to help them. They gave me the platform and the opportunity to address the people in the village gatherings … to tell them about what ASHAs are supposed to do, on what basis I am able to it … and what all I can help them with” *[ASHA XX Village].

 All ASHAs talked about the centrality of the support from village leaders to their overall experience of being an ASHA – often highlighting that without it, they would not be where they were.

###  Mobilising and Accumulating Social Capital 

 For the ASHAs, given their intermediary role between communities and the health system, the social context in which they worked, had a major bearing on how they experienced being a CHW. Questions about their status in society, how they were viewed within the village, and changes if any in social status on becoming an ASHA, elicited responses which illustrate the layered complexity of the subject. We found that improvement in social status was contingent upon apriori social status and position in the local social hierarchy. One ASHA, while recognizing that becoming and being an ASHA had enhanced her social status, explained that this would not be so for someone whose social status was already high. Her argument was that women from families with already high social status would not see any further enhancement in their status by becoming an ASHA.

 As the following interaction illustrates, on the contrary, becoming an ASHA might undermine such a person’s social status.

*“Interviewer: So, the village headman wouldn’t let his daughter-in-law become an ASHA? ASHA: His daughter-in-law … no way …it’s a matter of his prestige after all. How could a headman’s daughter-in-law be seen as going around from door to door? Everyone is conscious of their status in society” *[ASHA YY Village].

These findings imply that while becoming an ASHA could mean an enhancement in social status, it was only so for those who had a relatively low starting point. There is however another side to this. An experienced health worker pointed out that *“the daughters-in-law from affluent and high-status families do not come forward to become ASHAs,”* adding that *“those from poor families and from marginalized communities generally do not fulfil the recruitment criteria, so cannot become ASHAs”* [Health Worker].

 Many ASHAs confirmed this – indicating that ASHAs typically hail from families which are in the middle of the social hierarchy of the village; such families would be just prosperous enough that the daughter-in-law would not have to work as a farm labourer to support the family. The daughter-in-law would be involved in household activities or be working to some extent on the family’s own farms. The points raised above suggest that the act of opting to become an ASHA was often a very nuanced exercise of agency by the woman concerned. The decision entailed careful social calculations, striking a balance between the opportunities becoming an ASHA would open up for her personally, and the gains or risks to social status for herself and her family in doing so. We found the ASHAs to be a pragmatic lot. The above ASHA and all the others we interviewed, seemed to be saying “Sure … I know my station in life, but I am going to make the most of the hand that I have been dealt and improve my lot.”

During fieldwork we noticed that there were no ASHAs from poor and marginalized families. The one exception was an ASHA from YY village; and we found her to be barely hanging on to her job, and not particularly successful at it. It seemed that most of the women from poor and marginalized families who had started off as ASHAs, had quit. The experienced health worker referred to above explained that this was not surprising because *“For the poor women, the limited and unpredictable income linked to working as an ASHA is simply not enough … farm labour is much more lucrative and much more predictable” *[Health Worker]; many of the ASHAs we spoke with, concurred. While this situation may not be representative of ASHAs’ and CHWs’ experience at large, it is in many ways understandable given, as elaborated in the earlier section, and as illustrated in Box 3, working effectively as an ASHA does not leave much room for one to do regular income generating work. An alternative explanation also emerges from the earlier sections; Boxes 2 and 3 show how extensively an ASHA is required to mobilise social networks, social contacts and relational resources, to successfully fulfil her role. Those who are poor and marginalized do not have such social capital and are thus systematically disadvantaged in the successful performance of their roles, and in maintaining and advancing their careers. This insight spotlights a potentially important consideration for CHW programs as they scale up. At one level, CHW programs need to carefully assess who is able to become a CHW and who not; programs need to be careful that they do not contribute to reproducing and perpetuating entrenched social inequalities. At another level, programs can consciously support and enable the involvement of CHWs from poor and marginalized communities.

 Prima facie from the above account it appears that who becomes an ASHA (and who does not), mirrors the local social, power and gender relational arrangements – that it is merely a reproduction of the structural arrangements in the local society. However, the true picture was more complex; we found that ASHAs were fully aware of why they had been able to become an ASHA and what their families expected of them, and that their true exercise of agency lay in how they seized the opportunity that had come their way, and in how, many of them, as Boxes 2 and 3 showcase, made considerable effort to make the most of the opportunity. The following interaction with an ASHA from a relatively well-off family illustrates, albeit a bit awkwardly, this intentionality and resolve to work as an ASHA against all odds.

####  “Interviewer (I): You see … we have been speaking to many ASHAs. Respondent (R): Yes … I: Many say that unless one has one’s family’s support, working as an ASHA is not possible. R: Hmmm, indeed that is so. I: Is it so? [Long pause]. R: Well it is so with me too … [Long pause] … my husband is different … [Pause … some hesitation] … he doesn’t really work … [a brief but awkward silence] … he drinks (alcohol). I: Hmm … R: He talks … he berates me … he says why do I do this work … leave it … why do you have to work. I said … [nervous] … it’s my … my wish. I said I want to do it. I: OK. R: I said I won’t leave it (being ASHA). I said I have been working since 2007 … am I going to leave now? I am not going to leave. I: So, even when he says no … R: Yes, he says no … but I said I will absolutely not leave. I: So, you are doing this against his wishes. R: Yes [With steely resolve in her eyes], I am doing it” [ASHA YY Village]. 

 Not only does this excerpt present a compelling instance of exercise of agency by this ASHA, it spotlights how, perhaps for many women across India, the ASHA program offers a close to home platform and the means to challenge and subvert entrenched social and gender inequalities.

###  Exercise of Agency: Skilled, Pragmatic, and a Delicate Balancing Act

We observed that while ASHAs had a profound sense of relatedness and genuine concern for the communities they served, their accountability to the community was complicated. ASHAs were confronted with balancing 3 different accountability expectations and relations. *To their family*, as shown in the excerpt above, they had to continuously demonstrate the value proposition of them being allowed to step out of their homes, often at the expense of domestic chores. For many, this, as the following quote further illustrates, was an ongoing tough negotiation, which required persistence.

“ *Family members say to me... you work, but how much do you make? They say … you don’t have a salary … why do you do this work then? I say, I work because I want to. It is my desire to work … and I want to work whether I make money or not. I am still satisfied with what I get … I feel satisfied” *[ASHA YY Village].

Nevertheless, family pressure to bring a financial return pushed ASHAs to focus on those tasks which attracted the highest incentives^ [2],27^ and to pay much less attention to other, often equally important tasks. The balance was about paying just enough attention to the less rewarding tasks so as to prevent loss of credibility and legitimacy amongst community members and health staff.

The second accountability relationship was *with the health staff* at the local health facility and the sub-district level health system – their formal superiors in the health system. This accountability relationship was much more complicated than a mere supervisor-supervisee relationship. ASHAs exaggerated their expressions of accountability and demonstration of deference to the health staff – this was clearly an intentional and strategic enterprise. For one, this helped keep up the appearance of hierarchy within the ASHA-Health Staff relationship; this also allowed ASHAs to cultivate mutually beneficial arrangements with the local health facility staff. The staff would cooperate and facilitate the accrual of incentives to ASHAs, by often interpreting and/or documenting care events and care processes such that ASHAs would get more incentives; this included ASHAs being given credit by health workers for work they had not exactly done. We delicately queried this interpretation and application of the incentive arrangements, trying to gauge the risk of gaming of the incentive arrangements. Respondents openly but cautiously discussed it. They recognised that there was a fine line between propriety and flexible interpretation and application of the incentive arrangements; they insisted that this was done within what was allowed by the rules of the ASHA program, and was done transparently.

The third accountability relationship was *with the community* being served. ASHAs’ navigation of the first 2 accountability relationships on one hand, and the accountability expectations of the community members on the other, was remarkable. Accountability expectations of family and health staff very often did not align with those of community members. For instance – family members expected ASHAs to bring as much money home as possible; this expectation of bringing in money to one’s home aligned well with what the health staff expected (eg, greater service utilization; more institutional deliveries). However, what the community members expected and wanted, was different (eg, support with managing more common illnesses at home – for which there were no incentives for ASHAs). Similarly, the expectations of family members often did not align with those of health staff and community members. For instance, and again – family members expectations for ASHAs to bring as much money home as possible with as little time as possible spent outside the home, were inevitably at odds with what health workers expected (a hand always at their beck and call) or the community members wanted (someone available whenever they needed them). ASHAs’ ability to navigate these complex accountability relationships and expectations, keeping all parties just sufficiently satisfied to maintain their status, and to sustain the steady flow of incentives for themselves was demonstrative of a highly sophisticated and nuanced exercise of intentionality and agency.

 This delicate balancing act between competing imperatives, understandably often translated into ASHAs maximising their own benefits while maintaining only the minimally necessary modicum of accountability towards the beneficiary community. This finding and analysis however needs to be put in perspective and understood in terms of a key logic underpinning the occupation of public offices in the study area, and India broadly. Public offices are often considered a legitimate opportunity to accrue and exploit social, political and financial capital for the incumbents. The fact that ASHAs did not receive a salary, and their income depended on specific performance linked incentives, in some ways further justified the mobilization of this logic – by ASHAs, by their family members, their supervisors, health staff, and to some extent also community members.

## Discussion

The global health literature has extensively analysed the many ways in which CHWs’ actions, their performance, and their experiences are shaped by contextual forces – be they societal or organisational.^22,24^ The many problems experienced by CHWs because of their position at the bottom of the health system hierarchy, and because of the unequal gender power relations between the generally female CHWs and other health cadres and society, have been well documented,^23^ including in India.^19,20,28,29^ These issues are important and are material to our study context too – they need to be systematically and thoroughly addressed at the policy-practice level. Our study however sheds light on a hitherto under-reported aspect of being a CHW. We argue that the structural-functional explanatory accounts of CHWs’ experience and performance that dominate the literature are only partly accurate, and only partly complete. Our findings from the ASHA program in India reveal that CHWs may not be the mere rule takers at the bottom of the health system hierarchy, that they have often and historically been portrayed to be. In line with Van de Ruit’s^30^ insights from South Africa and Maes & Kalofonos’^11^ findings in Ethiopia, we show that CHWs are not weak and passive actors, but rather, skilled social actors with considerable tactical nous. The emphatic intentionality of ASHAs and their astute navigation of their complex context suggests that much can be gained through a closer look at CHW programs in other parts of the world. Maes and Kalofonos’s^11^ ethnographic work on CHWs in Ethiopia demonstrates the benefits of such an approach; as does Shet et al’s^31^ analysis of how lay CHWs in rural India exercise agency to deftly balance the competing demands on their time, in the face of little support from the health system. Our findings also echo and have implications for the discussions in the CHW field in some high-income countries.^32^ For instance, in the United States and Canada, CHWs are increasingly being called upon for frontline service delivery and are being integrated into healthcare systems.^32,33^ CHWs in these settings also face many of the same dilemmas as faced by CHWs in our study and in other LMIC contexts – dilemmas around how to navigate and negotiate entrenched relational arrangements and power dynamics in hierarchical health systems.

The ASHAs in our study in many ways embody Fligstein’s conceptualisation of the ‘skilled social actor.’^34^ Our findings show how the ASHAs “empathetically relate to the situations of other people and, in doing so, are able to provide those people with reasons to cooperate with each other” (p. 112). That they understand how various actors relate to each other, and where their interests lie. And how they “use this understanding in particular situations to provide an interpretation of the situation and frame courses of action that appeal to existing interests and identities,” (p. 112) inducing cooperation amongst a range of phenomena. Skilled actors are pragmatic and have deep understanding of what is possible and what not, and work with this understanding. Quite like the ASHAs in our study, a skilled actor will grab opportunities a situation might unexpectedly present, and will take “what the system will give at any moment, even if it is not exactly what the actor or others might ideally want” (p. 114) and make the most of it. We extend the research and policy-practice agendas on CHWs by demonstrating the value of explicitly accounting for agency, agentic potentials, and the accrual of social capital by CHWs. We argue that as CHW programs mature across the world, policy-makers and program managers need to remain alert to this accrual of social capital by CHWs, and to nurture their intentionality and agency; doing so will enable health systems to work more effectively with and better engage this important cadre. We show that even those who are putatively “at the bottom of the health system hierarchy,” do not merely act in reaction to structural powers, but wield agency, and over time, tacitly and explicitly shape the very structure they are embedded in.

 While demonstrating intentionality and exercise of agency by ASHAs, our findings also expose that this exercise of agency is riddled with many paradoxes. As workers at the bottom of the health system hierarchy, ASHAs are aware of their status; they experience and see the health system as a power that governs, monitors and regulates them. ASHAs also recognise that they are de facto agents and instruments of the health system – we realised that ASHAs clearly see the paradox inherent in this relational arrangement. The messy reality of ASHAs’ exercise of agency in associating with the health services and in mobilising social capital to enter into and to establish themselves as legitimate actors in complex relational arrangements, raise some knotty questions. Should this exercise of agency be seen as an expression and proof of ASHAs being able to successfully harness the resources and opportunities offered by the ASHA program and the government to simultaneously serve the health system, their community, and their family? Or does the exercise of agency to associate with the formal health services, to take up various activities of the ASHA program and the government, merely represent the process of ASHAs being absorbed into the lucrative spaces of governmentality and of ASHAs’ desires to climb the social ladder? How best should (and could) health programs engage with the explicit and tacit upending and subversion of entrenched social and gender hierarchies by ASHAs in the process?

While surely the reality is somewhere in between, our impression of the ASHAs in the study areas is that while ASHAs are explicitly complicit, they are tacitly subversive. In line with Mukhopadhyay’s^35^ argument, we see ASHAs’ attempts at assimilation into the governmental spaces, as a simultaneously complicit and subversive process. To us, this accrual of social capital and relational power by ASHAs connotes their active participation in spontaneous everyday politics to reconfigure their status in their relationship with the facility-based health workers representing the health system and the government.^36^ We found that over time, many ASHAs had successfully reconfigured the relationship such that they were no longer at the bottom of the hierarchy and were in some ways clearly equals of the more formal cadres at the primary care level. Our findings point to how CHWs, in the process of exercising agency to overcome the structural constraints they encounter while working at the bottom of the health system hierarchy, were actively interpreting their structural context, attaching unique meanings to their (and others’) situations, and shaping the very structural environment and powers that shaped their actions – reflecting what Appadurai^36^ calls “governmentality turned against itself.” These impressions are in line with Roalkvam^37^ who has also recognised this messiness of relationship dynamics between expectant mothers, ASHAs, the health system, and the broader society. They also accord with Shet and colleagues’ analysis^31^ of how in Karnataka, India, CHWs are improvising and collectivizing to reconfigure the very structural environment that has hitherto constrained them. The experiences of ASHAs in our study had similarities with what Mlotshwa et al^38^ report amongst CHWs in South Africa. For instance, ASHAs were also very aware of their multiple identities, and struggled with the tensions these created; the ASHAs’ work ethic was also underpinned by a spirit of humanity and kindness, similar to the notion of ‘ubuntu’ amongst the CHWs in South Africa. However, unlike the CHWs in South Africa, for many women, becoming an ASHA was perhaps the only way to have any meaningful identity of their own.

We found that the intentionality and exercise of agency by ASHAs was throughout foregrounded by the reality that being an ASHA and enacting the ASHA role occurred in a social realm where unequal and gendered power relations were entrenched and constantly operant, very often to the detriment of women. ASHAs were very aware of this, and communicated this awareness to us, both deliberately and unconsciously, across the 2 study sites. This clearly affirmed the salience of context; that this observation was true across the 2 study sites suggests that the differences in the economic contexts were not particularly relevant – the social and gender norms, and the institutional arrangements which were common across the 2 sites, were more material. At another level, and again common across the 2 study sites, the findings help put in perspective the tacit and explicit subversion of structural powers by ASHAs. Their actions were on one hand explicit pragmatic acts to achieve immediate instrumental ends. On the other hand, they were acts of resistance against the entrenched and gendered structural inequalities faced by ASHAs as women. The differences in the experiences and responses of ASHAs however, provide a reminder that one’s position in the social hierarchy and within one’s relational environment, determines one’s agentic possibilities. These findings echo and speak to feminist scholars’ contentions that assuming a universal, homogenous and autonomous agent, and not differentiating between the agentic potentials of agents (both within and between categories of agents), their place in society, and their ability (or not) and freedom (or not) to act, is problematic.^39-42^

Finally, in highlighting the complexity of being a CHW, our findings also demonstrate the value of being critical of the received wisdom, and alert to the constantly evolving power dynamics in complex social and health system relations. We argue that it is important to recognise that the process of exercising agency to navigate constraining contextual forces is a human enterprise which is vulnerable to human frailties and problems. These frailties have also been recognised by Van de Ruit^30^ in her analysis of how structural and local factors produced unintended consequences for CHW programs in South Africa. She shows how CHWs exploit the weaknesses of the health system that surveilles and regulates them, sometimes to the detriment of the communities they serve. Our findings that over years ASHAs/CHWs earn and accumulate substantial social capital and varying degrees of political capital also mean that they have some power within the community. While this was the case in both the study areas, it was best illustrated by the positive instance of an ASHA being recently elected as the head of the village panchayat. ASHAs/CHWs hail from the local community, have deep links within the community, have accumulated social capital and standing, have institutional memory, and have deep understanding of local social dynamics and politics. This creates conditions whereby they have some power over local health staff too. Health workers are civil servants and get transferred around to different locations in the state, and thus are not usually able to put down roots in the local community. ASHAs/CHWs on the other hand stay on in the village and serve as the eyes and ears of the health workers. In doing so, and in serving as the intermediary between communities and health facility staff, ASHAs have the power to steer and colour conversations and shape discourses; paradoxically, they have power over the very health workers they are subordinate to. This power is particularly potent given the growing trend towards greater citizen accountability and local political oversight of health services in India and globally.

In both the study areas we saw this power being exercised overtly and covertly, certainly to ASHAs’ own benefits, sometimes to the benefit of health workers, and to some extent to the benefit of the community being served. Further exploration and analyses are required to unpack this emerging relational dynamic, including in high income country health system contexts where similar observations have been made.^32^ Curiously, and in our view, also symbolic of the nuance and sophistication with which ASHAs exercised agency, across the board ASHAs assiduously cultivated and maintained a public persona of being someone who ‘serves’ and does so with great humility; they never acknowledged the power they had or potentially had. The choice of this public persona in the Indian context connotes the occupation of a certain local sociopolitical ground by the one who dons the persona – this is how many aspiring grassroot politicians would conduct themselves. To us it communicated a confident sense of self; the ASHAs seemed to be saying – “we have arrived.”

## Conclusion

 By highlighting the nuanced and deft exercise of agency by CHWs, we have shown that the time is right for health and social policy-makers to rethink and revisit how they view CHWs. A narrow and instrumentalist view of CHWs as merely an ‘extra pair of hands’ to be called upon to provide ‘technical fixes,’ clearly does injustice to the committed grassroots service many of them provide. Such an approach also ignores the vast social capital CHWs have and (may) accumulate over time – not acknowledging it and not engaging with it, is risky, and a missed opportunity for health systems. We argue that ASHAs, and CHWs generally, if appropriately enabled and supported, can be strong advocates of the disadvantaged and have the potential to make a strong contribution to equitable health development.

## Ethical issues

 The study was approved by the Independent Ethics Committee at Gokhale Institute of Politics & Economics, Pune, India.

## Competing interests

 Authors declare that they have no competing interests.

## Authors’ contributions

 SK conceptualised and led the study. SK, AR, and MG designed the tools, conducted the fieldwork and analysed the data. SK drafted the manuscript, AR, MG, and BM reviewed the manuscript and provided critical inputs.

## Authors’ affiliations

^1^Nossal Institute for Global Health, Melbourne School of Population and Global Health, The University of Melbourne, Parkville, VIC, Australia. ^2^Gokhale Institute of Politics and Economics, Pune, Maharashtra, India. ^3^State Health Systems Resource Centre, Pune, Maharashtra, India.

## Endnotes

 [1] Names of villages, sub-districts, and tertiary hospitals have been anonymised to ensure confidentiality. [2] ASHAs are remunerated through a task-performance based compensation arrangement. Incentives vary by task; some tasks carry high incentives (eg, INR 600 for an institutional delivery), while others attract very low (eg, INR 5 to facilitate a test for Sickle Cell Disease detection), or no incentives at all.


Supplementary file 1. Study Framework and Topic Guide.

Supplementary file 2. Profile of ASHAs Included in the Study.

## Key Messages

**Implications for policy makers**

- The tendency to view community health workers (CHWs) as merely an ‘extra pair of hands’ to be called upon to provide ‘technical fixes’ should be abandoned.
- As CHW programs in low- and middle-income country (LMIC) health systems evolve and mature, CHWs will accumulate substantial local social capital.
- Using the Indian health system as a case study, we show how CHWs in many instances are emerging as powerful social actors with influence in the communities they serve and also in frontline health services.
- It is critical that health systems recognise these emerging local social dynamics, appreciate their implications for frontline health service delivery, and leverage these as an opportunity to enhance health and social development.

**Implications for public**

In many low- and middle-income countries (LMICs), community health workers (CHWs) provide essential health services to underserved communities. CHWs tend to receive low salaries and are at the bottom of the health system hierarchy. A substantial body of research highlights the unfair treatment of CHWs, showing that they do not feel supported in their workplaces, and that they often find their work to be disempowering. This study, focused on CHWs in India reveals that the experience of being a CHW is evolving. We show that far from being powerless and weak as the literature tends to portray them, CHWs have the potential to emerge as powerful social actors with influence in the communities they serve and in the frontline health services. We argue that health policy-makers should take a more nuanced view of CHWs and engage with them as influential actors in their own right.
