# Supplementary material for: Community Health Workers as Influential Health System Actors and not "Just Another Pair Of Hands"
Source: Int J Health Policy Manag. 2020 Apr 27;10(8):465–74. doi: 10.34172/ijhpm.2020.58 (PMC9056200; doi:10.34172/ijhpm.2020.58)
Supplement: Supplementary file 1 — Study Framework and Topic Guide. [file ijhpm-10-465-s001.pdf]

Supplementary file 1. Study Framework and Topic Guide

Study Framework

Kane et al (2010) have postulated a set of seven mechanisms which explain community health workers' (CHWs) experience and performance. They have argued that people have a good experience of being CHWs and perform well if these mechanisms are triggered and vice versa. Drawing on evidence from CHW programs from different parts of the world, they demonstrate that the triggering (or not) of these mechanisms is influenced by the contextual conditions, and/or the CHW program design and/or implementation processes. These mechanisms have been widely recognised, directly (almost 100 citations) and indirectly (not always explicitly cited by, but these drivers of experience and performance are clearly recognisable). More recently, Druetz et al (2015) used this framework and the proposed mechanisms to examine the workplace experience and performance of community health workers in Burkina Faso. We also used these mechanisms as the starting point of our inquiry. The extant literature on CHWs/ASHAs work and performance, our knowledge of the local social and health system context, our experience of working in the study area, and these postulates, informed the inquiry (and the interview topic guides). The seven mechanisms are (adapted from Kane et al 2010):

- Sense of relatedness to the local public health services, and thus accountability to the system.
- Sense of credibility and legitimacy in being part of local public health services.
- Anticipation of being valued by local public health services.
- Assurance that there is a system for back-up support.
- Perception of improvement in social status and playing a valuable role.
- Sense of relatedness and accountability to the beneficiaries.
- Anticipation of being valued by the community.

Topic guide for interviews with ASHAs (modified for health workers)

Introductory steps and questions (begin after following: the consent processes, recording the cover sheet, and activating the recording device).

- Please tell me about yourself. (Inquire into the background - age, marital status, about family, hailing from, interests, what is important in life, children).

- I would like to hear about a typical day in your life. What occupies your time? What occupies your mind? Tell me about what you find most meaningful?
  (The purpose is to get to know her better and to understand her interests and her background as a person)
- Please tell me how you became an ASHA. Did you choose to become an ASHA? Was it easy? How would you describe the process (focus on the personal level experience – but listen if she describes the process)?
- Since when have you been an ASHA? Is someone else in your family/friends also an ASHA?

Substantive questions

- What does being an ASHA mean to you?
  - Probe to understand the meaning at a personal level.
  - Probe to understand the meaning at a social level.
  - Probe to understand why? Seek examples and explanations.
- How do people around you, look at you? View you? (way they see you) (compared to how it was before). Can you explain why this might have transpired?
  - Probe to understand why? Seek examples.

- How do you look at yourself? View yourself? (way you see yourself) (compared to how it was before). Can you explain why this might have transpired?
- As part of being an ASHA – Could you reflect upon what all shapes how people view you and look at you?
- Refer to earlier answer about self-image and ask - What all shapes and makes you look at yourself this way? (this is about trying to find out what influences ASHA's view of themselves, these should be probed at personal level, relational level and societal-structural level).
- You are from around here – isn't it?
- Do you think your standing in society has changed as a result of becoming an ASHA? Please give examples to explain and illustrate your claim.
- Please explain why you think this may have happened. What all-in your view might have contributed to this (this might need some probing to cover the things related to training

interventions, and maybe related to credibility and legitimacy possibly gained through association with the health services).

- Does this improved status (assuming that is the case) help you? A. To do your work as ASHA better? B. Socially? Seek examples which illustrate.
- This status is never easy to maintain – there are always things that come in the way (at work, in family, in society). Can you please reflect upon what all hinders and what all helps maintain or enhance this status?
    - Probe to arrive at explanations.
- Does this improved status, this bigger role weigh upon you as a responsibility? If so, in what ways? Please explain? If so, why does it weigh upon your mind?

Substantive questions continued

Let us look at your situation as someone working in relation with or together the Health Services. How do you see yourself in relation to the health services?

- How would you describe your relationship with the Health Services?
- What do you think is your role? Can you explain why you think so? (Probe for tasks that explain, contradict etc).
- Do you consider yourself as part of the health services?
- Do you think of yourself as a health worker like all other health workers at the Primary Health Centre and Health Post?
- Do you think of yourself as a public servant, as part of the health services?
- Do you think it is important that you are/be a part of the health services? Please explain.
- Do you think the community members see you as being part of the health services?
- Do you think it is important that you are seen as being a part of the health services? If so, by who all (probe for – formal health care providers, supervisors, villagers)? Seek explanations, and where appropriate, examples.
- Do you think the health workers working at the PHC, HP see you as health workers? As being part of the health services? (Probe for contradictions that they notice). Ask for explanations and examples of whatever they bring up.
- Who all-in the health services do you interact with? Probe for different relationships.

- With whom is the interaction most important? With whom is the interaction most difficult? With whom is the interaction the most satisfying? Seek explanations and examples.
- How would you like things to be?
    - So that you feel good generally (about yourself, about your work).
    - So that you can do your work better?

- Do you think ASHAs are valued by the system? Seek explanations and examples. Explore to what extent it matters (we do not assume that this is important to them).

Substantive questions continued

- Do you think ASHAs are valued by the system? Seek explanations and examples.
- Would you say that you are able to make a difference to the lives of your people?
    - In what ways?

- Would you say you could do more? (Probe for role beyond health)

Closure

- Ask if she would like to share anything that relates to what we have been talking about.
- Close by thanking her for her time, and for sharing her experiences.
- Reiterate that all that she has shared would be kept confidential.

References cited

Kane S, Gerretsen B, Scherpbier R, Dal Poz M, Dieleman M. A realist synthesis of randomised control trials involving use of community health workers for delivering child health interventions in low and middle-income countries. *BMC Health Serv Res*. 2010; Oct 13(10):286.

Druetz, T., Kadio, K., Haddad, S., Kouanda, S., & Ridde, V. Do community health workers perceive mechanisms associated with the success of community case management of malaria? A qualitative study from Burkina Faso. *Social Science & Medicine*. 2015; 124, 232-240.
