# Supplementary material for: Community Health Workers as Influential Health System Actors and not "Just Another Pair Of Hands"
Source: Int J Health Policy Manag. 2020 Apr 27;10(8):465–74. doi: 10.34172/ijhpm.2020.58 (PMC9056200; doi:10.34172/ijhpm.2020.58)
Supplement: Supplementary file 2 — Profile of ASHAs Included in the Study. [file ijhpm-10-465-s002.pdf]

Supplementary file 2. Profile of ASHAs Included in the Study

	Age	Education	Family Situation	Family (numbers and composition)	Years as ASHA	# of Children
1	NA	Grade 9	Widow living with two children	3 (Self, 2 Children)	1 yr	2
2	45	Grade 10	Nuclear Family	5 (Self, Husband and 3 Children)	11 yrs	3 (2 girls, 1 boy)
3	35	Grade 3	Nuclear Family	4 (Husband, Self and 2 Children)	8 yrs	3 (1 girl, 2 boys)
4	32	Grade 9	Joint Family	6 (Husband, Self, 2 Children, Father in Law and Mother in Law)	11 yrs	2 boys
5	35	Grade 6	Nuclear Family	5 (Husband, Self and 3 Children)	11 yrs	3 (2 girls, 1 boy)
6	33	Grade 12	Nuclear Family	3 (Husband, Self and 1 child)	9 yrs	1 boy
7	40	Grade 12	Joint Family	8 (Husband, Self, 4 Children, Father in Law and Mother in Law	9 yrs	4 (3 girls, 1 boy)
8	24	Grade 12	Joint Family	8 (Husband, Self, 2 Children, Father in Law and Mother in Law, Brother in Law and his Wife)	1 Year	2 (1 girl, 1 boy)
9	30	Middle School	Joint Family	8 (Husband, Self, 4 Children, Father in Law and Mother in Law)	11 yrs	4 (1 girl, 3 boys)
10	NA	Grade 12	Joint Family	8 (Husband, Self, 3 Children, Father in Law and Mother in Law, Brother in Law and His Wife)	11 yrs	3 (2 girls, 1 boy)
11	NA	Grade 8	Joint Family	10 (Husband, Self, 2 nephews, Father in Law and Mother in Law and Brother in Law and his Wife)	11 yrs	No Children
12	NA	Middle School	Nuclear Family	4 (Husband, Self, 2 Children)	8 yrs	2 girls
13	40	Grade 10	Joint Family	8 (Husband, Self, 2 Children, Father in Law and Mother in Law, Brother in Law and his Wife).	4 yrs	2 boys
14	20	Grade 8	Joint Family	7 (Husband, Self, 3 Children, Father in Law, and Mother in Law and Brother in Law)	3 yrs	1 girl child
15	26	Grade 7	Joint Family	6 (Husband, Self, 2 Children, Father in Law, and Mother in Law)	3 yrs	2 (1 girl, 1 boy)
16	25	Grade 8	Joint Family	7 (Husband, Self, 4 Children, and Mother in Law)	6 months	4 girls
17	32	Grade 10	Nuclear Family	4 (Husband, Self and 2 Children)	11 yrs	2 (1 girl, 1 boy)

Abbreviations: ASHA, Accredited Social Health Activists; NA, Not Available.
Many people in India are not sure of their age, as they do not know their dates/years of birth.
